# Association of In-hospital outcome of Acute Kidney Injury (AKI) with etiology among newborns at a tertiary care unit

**DOI:** 10.12669/pjms.341.13955

**Published:** 2018

**Authors:** Muhammad Asghar Ali, Abdur Rehman, Ejaz Ahmed

**Affiliations:** 1 Dr. Muhammad Asghar Ali, MBBS, FCPS (Pediatrics). Medical Officer, Department of Pediatric Neonatology, The Children's Hospital and Institute of the Child Health, Multan, Pakistan; 2 Dr. Abdur Rehman, MBBS, FCPS (Pediatrics), FCPS (Neonatology). Assistant Professor, Department of Pediatric Neonatology, The Children's Hospital and Institute of the Child Health, Multan, Pakistan; 3 Dr. Ejaz Ahmed, MBBS, FCPS (Pediatrics). Department of Pediatric Neonatology, The Children's Hospital and Institute of the Child Health, Multan, Pakistan

**Keywords:** Acute Kidney Injury, Sepsis, Etiology

## Abstract

**Objective:**

To determine association of in-hospital outcome of AKI with etiology in newborns at a tertiary care hospital.

**Methods:**

This descriptive cross-sectional study was conducted at Department of Pediatric Neonatology, The Children's Hospital and Institute of the Child Health, Multan by using non-probability purposive sampling technique from June 2016 to June 2017. A total of 101 newborns diagnosed with acute kidney injury were registered. Etiological factors were assessed and these patients were followed till discharge to monitor in-hospital outcomes.

**Results:**

Of these 101 newborns, 75 (74.3%) were boys while 26 (25.7%) were girls. Mean age of these newborns was 7.59 ± 6.13 days (range; 1 day to 28 days). Mean age of the boys was 5.73 ± 7.20 days while that of girls was 6.77 ± 6.16 days. (p=0.515). Mean weight of these neonates was 2545.05 ± 600.42 grams (range; 1000 grams to 4000 grams). Mean serum potassium level was 4.94 ± 0.92 mgEq/L ranging from 3.1 mgEq/L to 7.0 mgEq/L. Mean urea level was 73.35 ± 27.65 mg/dl ranging from 18 mg/dl to 206 mg/dl. Mean serum creatinine level was 1.98 ± 0.27 mg/dl, ranging from 1.6 mg/dl to 2.8 mg/dl. Mean serum sodium level was 145.72 ± 12.64 mgEq/L ranging from 126 to 166 mEq/L. Eighty one (80.2%) were term babies while 20 (19.8%) were pre-term babies. Of these 101 study cases, 29 (28.7%) delivered vaginally while 72 (71.3%) through cesarean section. Delayed crying was noted in 48 (47.5%), dehydration 13 (12.9%), sepsis in 36 (35.6%) and renal malformation in only 4%. Neonatal mortality in these patients was 15 (14.9%) while 86 (85.1%) were discharged from hospital after recovery.

**Conclusion:**

Acute kidney disease in newborns is associated with significant disease morbidity and mortality with asphyxia and sepsis are the main etiological factors responsible. It is predominantly more common in boys compared with girls. Mortality rate was high in our study and it was significantly associated with female gender. Mortality was also associated with elevated serum sodium and urea level.

## INTRODUCTION

Acute kidney injury (AKI), formerly referred to as acute renal failure (ARF), is defined as an acute reduction in kidney function that results in a decline in glomerular filtration rate (GFR) leading to retention of urea and other nitrogenous waste products, and loss of fluid, electrolyte, and acid-base regulation.^1^-^3^ AKI is an important contributing factor to the morbidity and mortality of critically ill neonates. Acute kidney injury refers to consistent increase in the levels of plasma creatinine by more than 1.5 mg/dl for more than 24 hours among full term newborns during their first few days of life provided that mother harbors normal kidney functions.^4^,^5^ Among preterm newborns, serum creatinine levels during their first days of life may not give a true picture of a reflection of the glomerular filtration rate (GFR) as its levels are usually high during first couple of days which then start decreasing gradually within first two weeks.^6^

Acute kidney injury, in different studies, has been reported to be ranging between 8% to 24% while these cases may further be classified in two groups i.e. Oliguric and Non-oliguric. However such reductions in urine output may also be seen in the absence of acute kidney injury, hence cannot be employed as sole criteria for the diagnosis.^7^,^8^

The cause of AKI among newborns remains to be multi-factorial, and generally there are multiple related contributing agents associated with AKI in neonates. In majority of cases birth asphyxia and sepsis are commonly encountered underlying conditions of AKI in these patients while other conditions in neonates associated with development of AKI may be; dehydration, bleeding, respiratory distress syndrome (RDS), congestive cardiac failure (CCF) and nephrotoxic drug.^9^,^10^

Among newborns common etiological factors include “Congenital malformations (including renal dysplasia, hypoplasia, agenesis and renal cysts); acquired kidney diseases including acute tubular necrosis, vascular events (renal artery or vein thrombosis), or medications (angiotensin-converting enzyme inhibitor or indomethacin usage during pregnancy); and obstructive uropathy”.^9^,^10^

Estimation of serum creatinine levels remains the simplest, robust and widely adopted means for the assessment of kidney functions which drops significantly from 1.1 mg/dl to 0.4 mg/dl during first two weeks of life following term delivery while from 1.3 mg/dl in case of preterm infants.^11^-^13^ Treatment options may include conservative management, dialysis and surgical interventions in case of obstructions in urinary tract while peritoneal dialysis is a treatment of choice compared with other dialysis procedures particularly among low birth weight newborns.^14^,^15^ The onset AKI may also be prenatal congenital disease including renal dysplasia, obstructive uropathy and autosomal recessive polycystic kidney disease while it is commonly acquired in postnatal periods as a result of hypoxic ischemic injuries as well toxic insult. Nephrotoxic AKI in generally associated with use of aminoglycoside antibiotics and nonsteroidal anti-inflammatory drugs which are used for closure of patent ductus arteriosis while some studies have also reported genetic risk factors of acute renal failure among neonates.^16^

This study was done to determine association of outcome of AKI with etiological factors of acute kidney injury (AKI) among newborns of Southern Punjab, Pakistan owing to the scarcity of local data on this topic.

## METHODS

This study was done at Neonatal Intensive Care Unit (NICU) of the Children's Hospital and institute of the Child Health, Multan which provides tertiary care level facilities to the population of 35 million people of Southern Punjab and associated areas of Sindh, Balochistan and Khyber Pakhtunkhawah. A total of 101 newborns with Acute Kidney Injury admitted to the NICU of our hospital were included in this cross-sectional study.

Sample size was calculated by sample size calculator of Epi-info software of CDC by anticipating 20%^17^ mortality rate among newborns with AKI i.e. (p=20%), margin of error was 8% among newborns with AKI Newborns of either sex less than 28 days having acute kidney injury were included in this study. AKI was defined as serum creatinine levels more than 1.5 mg/dl irrespective of the age. The newborns were clinically assessed by a consultant pediatrician for different causes of the AKI such as dehydration, sepsis, asphyxia neonatorum and renal malformation. Dehydration was defined by presence of any two of the following conditions; Sunken eyes, dry mucus membrane, having depressed fontanelle, unconsciousness, lethargy, heart rate more than 160 per minute (tachycardia) and hypotension defined as having systolic blood pressure less than 60 mm Hg. Neonatal sepsis was defined as serum C – reactive protein levels more than 6 mg/dl plus any two of the following conditions; Temperature instability hypothermia characterized as less than 35 C° or hyperthermia (more than 38.5 °F), tachycardia defined as patient having heart rate 160 per minute, delayed capillary refill time more than three seconds, tachypnea > 60/ minute. Asphyxia neonatorum was defined as if newborn failed to initiate and sustain breathing after 60 seconds of cutting umbilical cord and is related with delayed crying for more than five minutes. Renal malformation was diagnosed ultrasonographically revealing morphological defects which was reported by a senior Sonologist having more than 10 years of relevant experience. Outcome of AKI in newborns was measured in terms mortality during current hospitalization. Other relevant information like age, gender, gestational age and mode of delivery were also noted in the pre-designed study proforma.

**Table-I:** Cross – tabulation of in-hospital outcome with study variables. (n=101)

Variables		Outcome		P–value

		Discharged	Death	
Gender	Boys (n=75)	67	08	0.05
	Girls (n=26)	19	07	
	Term (n=81)	70	11	
Gestation	Preterm (n=20)	16	04	0.489
Mode of delivery	Vaginal (n=29)	26	03	0.545
	Cesarean section (n=72)	60	12	
Delayed Crying	Yes (n=48)	41	07	1.000
	No (n=53)	45	08	
Sepsis	Yes (n=36)	32	04	0.564
	No (n=65)	54	11	
Dehydration	Yes (n=13)	10	03	0.404
	No (n=88)	76	12	
Renal malformation	Yes (n=04)	03	01	0.480
	No (n=97)	83	14	

Data obtained was entered in SPSS version 16 on the computer to analyze mean and standard deviations for the numerical study variables like age (in days), serum creatinine levels (in mg/dl), Serum sodium levels. Gender, mode of delivery, gestation, dehydration, sepsis, delayed crying, renal malformation and outcome (discharged/expired) were tabulated in terms of frequencies and percentages. Outcome was cross-tabulated against gender, gestation, mode of delivery, delayed crying, sepsis, renal malformation, dehydration and chi-square test was applied to see their impact on outcome while for numerical variables of the study independent sample t test was used at level of significance of 0.05.

**Table-II:** Serum Biochemical parameters with regards to in-hospital outcome. (n=101)

Biochemical parameters		Outcome		P–value

		Discharged	Death	
Serum Potassium Level (mEq/L)	Mean	4.91	5.12	0.437
	SD	0.86	1.22	
Serum Urea (mg/dl)	Mean	69.35	96.27	0.001
	SD	23.67	37.45	
Serum Sodium level (mEq/L)	Mean	144.62	152.00	0.037
	SD	12.90	9.05	

## RESULTS

We recruited a total of 101 newborns with acute kidney injury. Of these 101 newborns, 75 (74.3%) were boys while 26 (25.7%) were girls. Mean age of these newborns was 7.59 ± 6.13 days (range; 1 day to 28 days). Mean age of the boys was 5.73 ± 7.20 days while that of girls was 6.77 ± 6.16 days. (p=0.515). Mean weight of these neonates was 2545.05 ± 600.42 grams (range; 1000 grams to 4000 grams). Mean serum creatinine level was 1.98 ± 0.27 mg/dl, ranging from 1.6 mg/dl to 2.8 mg/dl. Mean serum potassium level was 4.94 ± 0.92 mgEq/L ranging from 3.1 mgEq/L to 7.0 mgEq/L. Mean urea level was 73.35 ± 27.65 mg/dl ranging from 18 mg/dl to 206 mg/dl. Mean serum sodium level was 145.72 ± 12.64 mgEq/L ranging from 126 to 166 mEq/L. Eighty one (80.2%) were term babies while 20 (19.8%) were pre-term babies. Of these 101 study cases, 29 (28.7%) delivered vaginally while 72 (71.3%) through cesarean section. Delayed crying was noted in 48 (47.5%), dehydration 13 (12.9%), sepsis in 36 (35.6%) and renal malformation in only 4%. Neonatal mortality in these patients was 15 (14.9%) while 86 (85.1%) were discharged from hospital after recovery.

**Table-III:** Serum Biochemical parameters with regards to delayed crying. (n=101)

Biochemical parameters		Delayed Crying		P–value

		Yes	No	
Serum Potassium Level (mEq/L)	Mean	5.18	4.73	0.014
	SD	0.83	0.94	
Serum Urea (mg/dl)	Mean	79.69	67.66	0.028
	SD	42.46	21.17	
Serum Sodium level (mEq/L)	Mean	146.79	144.75	0.422
	SD	9.41	15.02	

**Table-IV:** Serum Biochemical parameters with regards to sepsis. (n=101)

Biochemical parameters		Sepsis		P–value

		Yes	No	
Serum Potassium Level (mEq/L)	Mean	4.52	5.18	0.001
	SD	0.96	0.80	
Serum Urea (mg/dl)	Mean	66.03	77.40	0.047
	SD	18.35	31.06	
Serum Sodium level (mEq/L)	Mean	145.13	146.04	0.732
	SD	9.15	14.27	

**Table-V:** Serum Biochemical parameters with regards to dehydration. (n=101)

Biochemical parameters		Dehydration		P–value

		Yes	No	
Serum Potassium Level (mEq/L)	Mean	5.30	4.89	0.702
	SD	0.68	0.93	
Serum Urea (mg/dl)	Mean	76.00	72.95	0.070
	SD	26.06	28.01	
Serum Sodium level (mEq/L)	Mean	150.84	144.96	0.017
	SD	6.61	13.16	

## DISCUSSION

Different studies reported from various parts of the world have documented high prevalence of predisposing factors of acute renal failure in boys as compared with girls. Similarly in our study there were 75 (74.3%) boys while 26 (25.7%) were girls. Gharehbaghi et al.^17^ from Iran also reported male to female ratio was 2.03:1 (67% versus 33%) showing same trends as that of our study results. Similar results have been reported by Airede et al.^18^ Kandoth et al.^19^ and Bourquia et al.^20^ However Momtaz et al.^21^ have reported different findings showing female gender predominating among newborns with AKI. Acute renal failure is generally observed in first few days of life to couple of weeks time. Similarly in our study mean age of the newborns with AKI was 7.59 ± 6.13 days (range; 1 day to 28 days). Mean age of the boys was 5.73 ± 7.20 days while that of girls was 6.77 ± 6.16 days. (p=0.515). Gharehbaghi et al.^17^ from Iran also reported 5.26 ± 6.2 days ranging from 2–28 days. Similarly, Momtaz et al.^21^ reported 7.7 ± 6.3 days mean age in newborns with AKI. Mean weight of these neonates was 2545.05 ± 600.42 grams (range; 1000 grams to 4000 grams). Gharehbaghi et al.^17^ from Iran also reported2682.58 ± 629.33 mean weight, close to our results.

In our study 80.2% were term babies while 19.8% were pre-term babies. Similarly, Gharehbaghi et al.^17^ from Iran also reported 25.9% preterm deliveries. Momtaz et al.^21^ documented 20.5% prematurity in newborns with acute renal failure.

Birth asphyxia and sepsis have been reported to be associated significantly with AKI in newborns all over the world^21^-^23^ which may reach as higher as 78% in some studies. Delayed crying and sepsis were noted 47.5% and 35.6% respectively. Airede et al.^18^ and Gharehbaghi et al.^17^ also reported similar results. High mortality rates have already been reported by different authors in children suffering from acute renal failure, in our study mortality rate was 14.9%. Gharehbaghi et al.^17^ from Iran also reported 20% mortality rate. In our study mortality was significantly higher in girls which is in compliance to similar results reported by Gharehbaghi et al.^17^ Momtaz et al.^21^ reported 36.7% mortality rate and also documented its association with female gender. However the reasons for this significant association of mortality in girls are not yet known. Similarly serum urea and serum sodium levels were also significantly higher in newborns with mortality. Other studies have documented sepsis as an underlying cause of mortality in these newborns, however our study results show different trends as this association was not statistically significant.

## CONCLUSION

Acute kidney disease in newborns is associated with significant disease morbidity and mortality with asphyxia and sepsis are the main etiological factors responsible. It is predominantly more common in boys compared with girls. Mortality rate was high in our study and it was significantly associated with female gender. Mortality was also associated with elevated serum sodium and urea level.

### Authors' Contribution

**MAA:** Conceived idea, study planning and designing, manuscript writing and data collection.

**AR:** Supervised the research work, editing and proof reading of the manuscript and study planning.

**EA:** Data entry, data analysis, data collection and manuscript editing.
